# Renal function damage in children with duplex kidneys

**DOI:** 10.1007/s11255-022-03337-8

**Published:** 2022-09-14

**Authors:** Qian Lin, Jiajia Ni, Yufeng Li, Jing Jin, Yaju Zhu

**Affiliations:** 1 Pediatric College, Shanghai Jiao Tong University School of Medicine, Shanghai, 200092 China; 2 Department of Pediatric Nephrology, Rheumatology and Immunology, Xinhua Hospital Affiliated to Shanghai Jiaotong University School of Medicine, No. 1665, Kongjiang Road, Yangpu District, Shanghai, 200092 China

**Keywords:** Duplex kidney, Split renal function, Renal dynamic imaging, Children, Vesicoureteral reflux, Dimercaptosuccinic acid

## Abstract

**Purpose:**

To evaluate renal function damage in children with duplex kidneys.

**Methods:**

A total of 355 duplex kidneys, 110 urinary tract infection (UTI) kidneys without abnormalities, and 104 kidneys with primary unilateral vesicoureteral reflux (VUR) were reviewed. Clinical data including age at diagnosis, body weight, history of UTI, ureteroceles, ectopic ureteral opening, VUR grade, serum creatinine level, cystatin C level, renal scarring, split renal function in dimercaptosuccinic acid scans, and effective renal plasma flow (ERPF) were analyzed.

**Results:**

Duplex kidneys had a higher grade of VUR and renal scarring. Split renal function in unilateral duplex kidneys (45.58 ± 12.85%) was much lower than that in contralateral duplex kidneys (56.33 ± 11.90%) and controls (50.00 ± 11.38%) (*P* < 0.001 and *P* = 0.014, respectively). Both left and right split renal functions in bilateral duplex kidneys were similar to those ipsilateral to the controls (*P* = 0.906 and *P* = 0.932, respectively). However, the total ERPFs in the left, right, and bilateral duplex kidneys were significantly lower than that in the control group (*P* = 0.003, *P* = 0.001, and *P* = 0.003, respectively). The total ERPFs in the left and right unilateral duplex kidneys were similar. ERPF in unilateral duplex kidneys (106.70 ± 48.05 mL/min/m^2^) was significantly lower than that in contralateral duplex kidneys (150.18 ± 49.01 mL/min/m^2^) or those ipsilateral to controls (145.98 ± 41.16 mL/min/m^2^) (*P* < 0.001 and *P* < 0.001, respectively).

**Conclusion:**

Duplex kidneys are usually accompanied by a higher grade of VUR, more severe renal scarring, and renal function impairment. Split renal function in duplex kidneys often declines significantly. Notably, the evaluation of split renal function in bilateral duplex kidneys should be performed cautiously.

## Introduction

Congenital anomalies of the kidney and urinary tract (CAKUT), including renal hypoplasia, renal dysplasia, vesicoureteral reflux (VUR), neurogenic bladder, and duplex kidney, are among the main causes of chronic kidney disease in children. A duplex kidney refers to the existence of two sets of collecting systems in the kidney on the affected side of the urinary system, usually resulting in upper moiety dysplasia and dominant development of the lower moiety. Duplex kidneys are often accompanied by ureteroceles, ectopic ureteral opening, VUR, and other malformations [1, 2]. Ectopic ureteral opening and VUR often cause recurrent urinary tract infection (UTI), and recurrent UTIs are high-risk factors for renal scar formation and kidney function damage [3]. The function of the duplex kidney must be assessed early. Dimercaptosuccinic acid (DMSA) renal scintigraphy provides an accurate assessment of functional renal parenchyma [4], and dynamic renal scintigraphy provides an accurate assessment of split renal function in hydro-nephrosis [5]. ^99m^Technetium-ethylene-dicysteine (^99m^Tc-EC) dynamic renal scintigraphy is beneficial for evaluating transplant kidney function [6].

The purpose of this retrospective study was to evaluate renal damage in children with duplex kidneys by comparing radionuclide images.

## Patients and methods

### Clinical data collection

The study included children aged ≤ 12 years who underwent treatment at a single tertiary care center between January 2015 and December 2020. All children with duplex kidneys were screened. Duplex kidneys were diagnosed using ultrasonography or DMSA scanning, and original records were reviewed. Age at diagnosis, sex, body weight, history of UTI, ureteroceles, ectopic ureteral opening, VUR grade, serum creatinine level, serum cystatin C level, DMSA scan, and effective renal plasma flow (ERPF) values were retrospectively analyzed. Ultrasound, voiding cysto-urethrography (VCUG), DMSA scanning, and dynamic renal scintigraphy were performed within 6 months. The ERPF values of split renal function were corrected by body surface area, which was calculated according to the following formula: body surface area = 0.035 × body weight (kg) + 0.1 m^2^ [7]. Children with UTI without urinary tract abnormalities and primary unilateral VUR during the same period were included in the control group.

### Definitions

Febrile UTI: The diagnosis of febrile UTI was based on a positive dipstick result for leukocyte esterase or nitrite and a significant bacterial count of a pure colony (≥ 10^5^ cfu/mL in the clean catch specimen, ≥ 10^4^ cfu/mL in the catheter, or any growth in bladder aspiration) in children presenting with a fever of > 38 °C.

Recurrent UTI: Recurrent UTI was defined as at least three episodes of UTI in 12 months or at least two episodes in 6 months.

### VUR grade

VCUG is the “gold standard” technique for the detection of VUR. It provides high-resolution anatomical images of the renal parenchyma, calyx, pelvis, and bladder. The ureters and urethra were partially visualized. The diagnosis of VUR was made by demonstration of reflux of urine into the upper urinary tract by conventional VCUG. VUR grading was performed according to the International Reflux Study Group classification [8].

### Renal parenchymal defects

Renal scarring on DMSA scans was defined as decreased uptake with loss of contours or cortical thinning with distortion of parenchymal volume. Renal damage was characterized as focal (single delimited area with decreased uptake), multifocal (more than one uptake defect), or generalized (small kidney with generalized reduced tracer uptake). Renal scarring was scored as focal = 1, multifocal = 2, generalized = 3, or no scarring = 0.

### Dynamic renal scintigraphy

Patients were administered 10–20 mL/kg of water orally 30–40 min before the procedure. The posterior dynamic acquisition was performed after intravenous injections of 3.7 MBq/kg of body weight of ^99m^Tc-EC and 1 mg/kg of body weight of furosemide (with a maximum of 20 mg). Images were processed by an independent senior nuclear medicine physician using a workflow software (Philips, Amsterdam, Netherlands). Regions of interest were manually drawn on the kidneys, heart, and C-shaped peri-renal background. The relative function was determined using the Patlak–Rutland method or the area under the curve method in studies in which the cardiac curve did not meet optimum quality, according to the international consensus recommendation [5]. Drainage was quantitatively assessed by normalized residual activity (NORA), renal output efficiency, and T_max_.

### Statistical analysis

Data are expressed as mean ± standard deviation. One-way ANOVA, *t* test, and Pearson’s *χ*^2^ test were used to test the measurement data of the two groups. Statistical analyses were performed using the SPSS software (version 19.0; SPSS, Chicago, IL, USA). A two-sided *P* value of < 0.05 was considered statistically significant.

## Results

### General information

A total of 317 children with duplex kidneys were eligible for inclusion in this study. A duplicated system was present in 355 kidneys bilaterally in 38 cases, right-sided in 107 cases, and left-sided in 172 cases. During the same period, 55 hospitalized children with UTIs without urinary tract abnormalities and 104 children with primary unilateral VUR (65 cases with left and 39 with right VUR) were included in the control group. Age at diagnosis, sex ratio, body weight, ureteroceles, and history of UTI showed no significant differences among the unilateral, bilateral, and control groups (Table 1). The ectopic ureteral opening was more common in the left or right duplex kidney than in the bilateral duplex kidney (*P* = 0.011 and *P* = 0.042, respectively) (Table 1).

**Table 1:** Clinical characteristics and renal function in different groups

	Left duplex kidney (*n* = 172)	Right duplex kidney (*n* = 107)	Bilateral duplex kidney (*n* = 38)	Control (*n* = 55)	*F* value	*P* value
Age at diagnosis (months)	17.82 ± 27.39	18.40 ± 21.69	17.01 ± 26.01	23.67 ± 25.33	0.828	0.479
Body weight (kg)	10.99 ± 8.07	10.86 ± 5.21	10.49 ± 5.80	12.37 ± 7.66	0.734	0.532
Gender (boy, %)	45 (26.16%)	24 (22.43%)	10 (26.32%)	22 (40.00%)	5.879	0.118
Recurrent UTI (*n*, %)	67 (38.95%)	44 (41.12%)	20 (52.63%)	25 (45.45%)	2.715	0.438
Febrile UTI (*n*, %)	96 (55.81%)	56 (52.34%)	17 (44.74%)	36 (65.45%)	4.387	0.223
Cystatin C (mg/L)	0.90 ± 0.24	1.00 ± 0.30	1.20 ± 0.42	0.78 ± 0.26	8.325	< 0.001
Serum creatinine (umol/L)	24.48 ± 8.43	25.97 ± 7.48	24.47 ± 7.81	24.08 ± 7.56	0.974	0.405
Ureteroceles (*n*, %)	78 (45.35%)	52 (48.60%)	31 (40.79%)		1.090	0.580
Ectopic ureteral opening (*n*, %)	67 (38.95%)^a^	39 (36.45%)^b^	17 (22.37%)		6.604	0.037
VCUG	*n* = 130	*n* = 76	*n* = 33	*n* = 55		
VUR (left)						
Mild (I, II)	5 (3.85%)	3 (3.95%)	2 (6.06%)	0.00	0.336	0.845
Moderate (III)	8 (6.15%)	2 (2.63%	5 (15.15%)	0.00	6.113	0.047
Severe (IV, V)	24 (18.46%)	3 (3.95%)	7 (21.21%)	0.00	9.770	0.008
VUR (right)						
Mild (I, II)	5 (3.85%)	1 (1.32%)	4 (12.12%)	0.00	6.754	0.034
Moderate (III)	2 (1.54%)	5 (6.58%)	3 (9.09%)	0.00	5.316	0.070
Severe (IV, V)	2 (1.54%)	19 (25.00%)	5 (15.15%)	0.00	27.834	< 0.001
DMSA						
Left kidney						
RR, %	45.22 ± 11.90	59.36 ± 11.80	49.87 ± 14.80	49.42 ± 11.43	6.636	< 0.001
Renal scarring	0.76 ± 0.95	0.29 ± 0.72	0.92 ± 0.95	1.18 ± 0.75	5.597	0.001
No scarring (*n*)	25	21	5	13		
Focal (*n*)	15	2	5	23		
Multifocal (*n*)	9	3	2	18		
Generalized (*n*)	3	0	1	0		
Right kidney						
RR,%	54.93 ± 11.86	40.64 ± 11.80	50.90 ± 14.19	50.58 ± 11.43	6.879	< 0.001
Renal scarring	0.39 ± 0.80	1.10 ± 0.89	0.85 ± 0.90	0.91 ± 0.76	4.887	0.003
No scarring (*n*)	41	7	5	21		
Focal (*n*)	4	10	6	24		
Multifocal (*n*)	6	7	1	8		
Generalized (*n*)	1	2	1	1		
Dynamic renal scintigraphy						
Total ERPF	255.85 ± 78.73	247.70 ± 80.12	243.33 ± 76.18	291.89 ± 60.05	4.760	0.003
Left kidney						
ERPF (left)	104.75 ± 43.51	148.16 ± 47.30	119.01 ± 57.62	144.43 ± 40.05	23.889	< 0.001
RR (left) %	40.63 ± 10.42	60.68 ± 10.10	49.71 ± 19.60	49.63 ± 10.57	65.866	< 0.001
Right kidney						
ERPF (right)	151.43 ± 50.14	99.54 ± 45.58	123.44 ± 58.83	147.54 ± 42.56	27.066	< 0.001
RR (right)%	59.49 ± 10.38	39.33 ± 10.10	50.00 ± 19.56	50.40 ± 10.40	67.204	< 0.001

*UTI* urinary tract infection, *VCUG* voiding cysto-urethrography, *VUR* vesicoureteral reflux, *DMSA* dimercaptosuccinic acid, *ERPF* effective renal plasma flow, focal renal scarring: single delimited area with decreased uptake; multifocal renal scarring: more than one uptake defect; generalized renal scarring: a small kidney with generalized reduced tracer uptake; *NA* no data

^a^Compare with bilateral duplex kidney (*P* = 0.011)

^b^Compare with bilateral duplex kidney (*P* = 0.042)

### Duplex kidneys accompanied by more renal scarring and a higher grade of VUR

The renal scarring scores in the left duplex kidney and right duplex kidney were 0.76 ± 0.95 and 1.10 ± 0.89, respectively, while those on the contralateral side were 0.39 ± 0.80 and 0.29 ± 0.72, respectively (Table 1). The renal scarring score in the unilateral duplex kidney was higher than that on the contralateral side (*P* < 0.001) (Table 2). The renal scarring score in the unilateral duplex kidney (0.91 ± 0.93) was similar to that of the ipsilateral kidney in controls (0.94 ± 0.77) (*P* = 0.784) (Table 2). The split renal scarring score in the reflux duplex kidney (1.18 ± 1.00) was higher than that in the duplex kidney (0.91 ± 0.93) (*P* = 0.035) (Table 2).

**Table 2:** Split renal function, renal scarring and VUR grade in duplex kidneys

	Unilateral duplex kidney (*n* = 355)	Contralateral duplex kidney (*n* = 279)	Unilateral reflux kidneys (*n* = 104)	Ipsilateral kidney in control (*n* = 110)	*F* value	*P* value
VCUG	*n* = 272	*n* = 206	*n* = 104	*n* = 110		
VUR grade						
Mild (I, II)	12 (4.41%)	8 (3.88%)	21 (20.19%)	0.00	4.856	0.088
Moderate (III)	21(7.72%)	4(1.94%)	35 (33.65%)	0.00	15.598	< 0.001
Severe (IV, V)	55(20.22%)	5 (2.43%)	48 (46.15%)	0.00	55.785	< 0.001
DMSA scan	*n* = 94	*n* = 68	*n* = 73	*n* = 90		
Split renal scarring score	0.91 ± 0.93^a^	0.35 ± 0.74	1.18 ± 1.00^b^	0.94 ± 0.77	13.621	< 0.001
Split renal function (%)	45.58 ± 12.85^c^	56.33 ± 11.90	39.52 ± 11.93	50.00 ± 11.38	15.612	< 0.001
Dynamic renal scintigraphy	*n* = 355	*n* = 279	*n* = 83	*n* = 110		
ERPF (ml/min/m^2^)	106.70 ± 48.05^d^	150.18 ± 49.01^e^	105.80 ± 65.26	145.98 ± 41.16	73.945	< 0.001
Split renal function (%)	42.21 ± 13.39	59.94 ± 10.28	35.92 ± 13.87	50.01 ± 10.44	173.781	< 0.001

*VCUG* voiding cysto-urethrography, *VUR* vesicoureteral reflux, *DMSA* dimercaptosuccinic acid, *ERPF* effective renal plasma flow, *n* cases

^a^Compare with contralateral side of duplex kidney (*P* < 0.001)

^b^Compare with unilateral duplex kidney (*P* = 0.035)

^c^Compare with reflux kidneys (*P* = 0.001)

^d^Compare with contralateral side of duplex kidney (*P* < 0.001)

^e^Compare with unilateral duplex kidneys (*P* < 0.001)

The total ratio of VUR in duplex kidneys was 32.35%. The ratios of moderate and severe VUR grades in the unilateral duplex kidney were 7.72% and 20.22%, respectively, which were significantly higher than those in the contralateral kidney of 1.94% (*P* < 0.001) and 2.43% (*P* < 0.001), respectively. The ratios of moderate and severe VUR grades in the unilateral reflux kidney were significantly higher than those in the contralateral and unilateral duplex kidneys (*P* < 0.001 and *P* < 0.001, respectively) (Table 2).

### Total renal function in duplex kidney

**Fig. 1:**
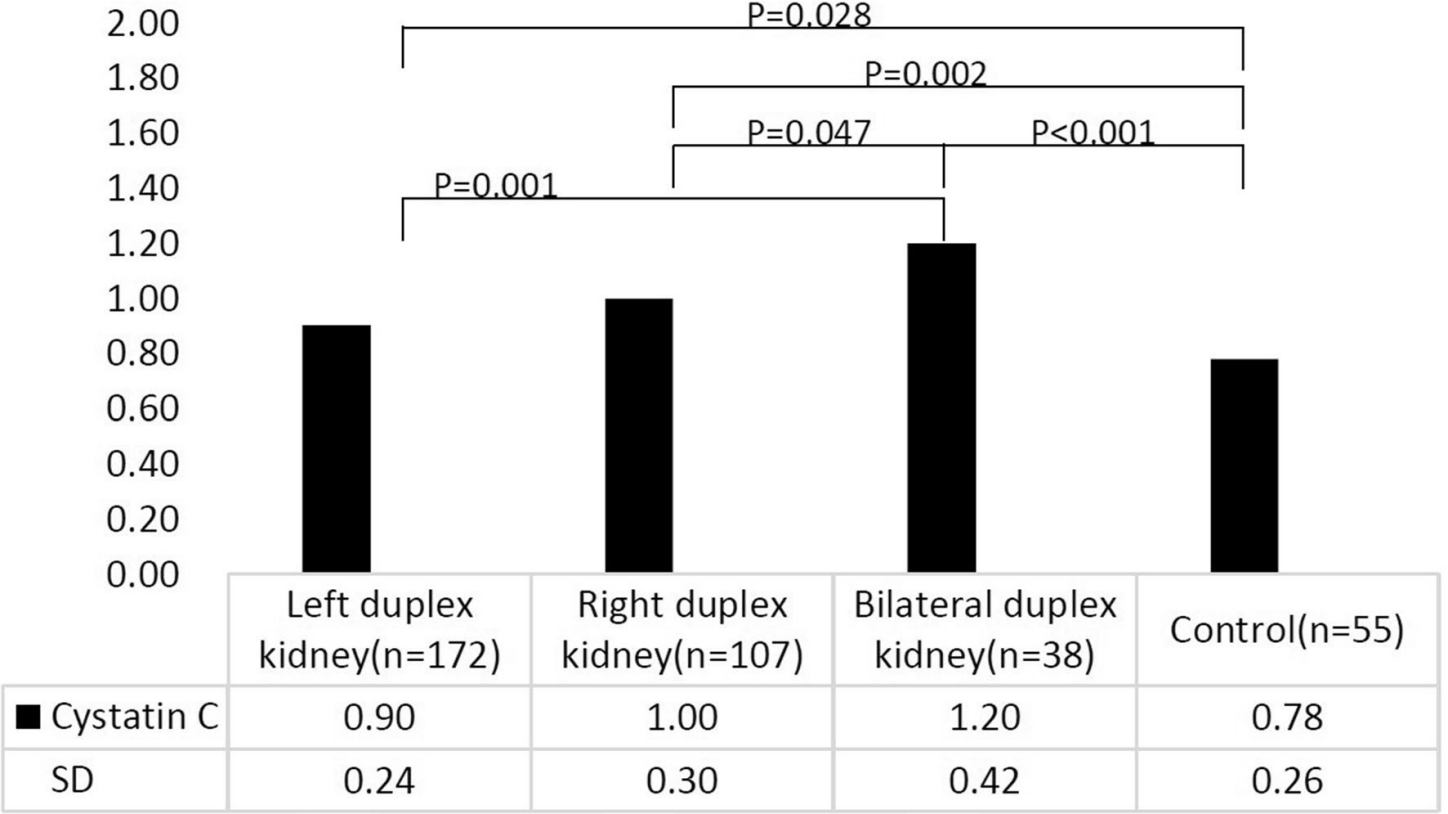
Serum cystatin C level in duplex kidney

The serum creatinine values in the left duplex kidney, right duplex kidney, bilateral duplex kidney, and control group were 24.48 ± 8.43 umol/L, 25.97 ± 7.48 umol/L, 24.47 ± 7.81 umol/L, and 24.08 ± 7.56 umol/L, respectively. There were no significant differences among the groups (*P* = 0.405) (Table 1). The serum cystatin C values in the left duplex kidney, right duplex kidney, bilateral duplex kidney, and control group were 0.90 ± 0.24 mg/L, 1.00 ± 0.30 mg/L, 1.20 ± 0.42 mg/L, and 0.78 ± 0.26 mg/L, respectively. The serum cystatin C levels in children with bilateral duplex kidneys were much higher than those in children with unilateral duplex kidneys and control children (*P* = 0.001, *P* = 0.047, and *P* < 0.001, respectively) (Fig. 1).

**Fig. 2:**
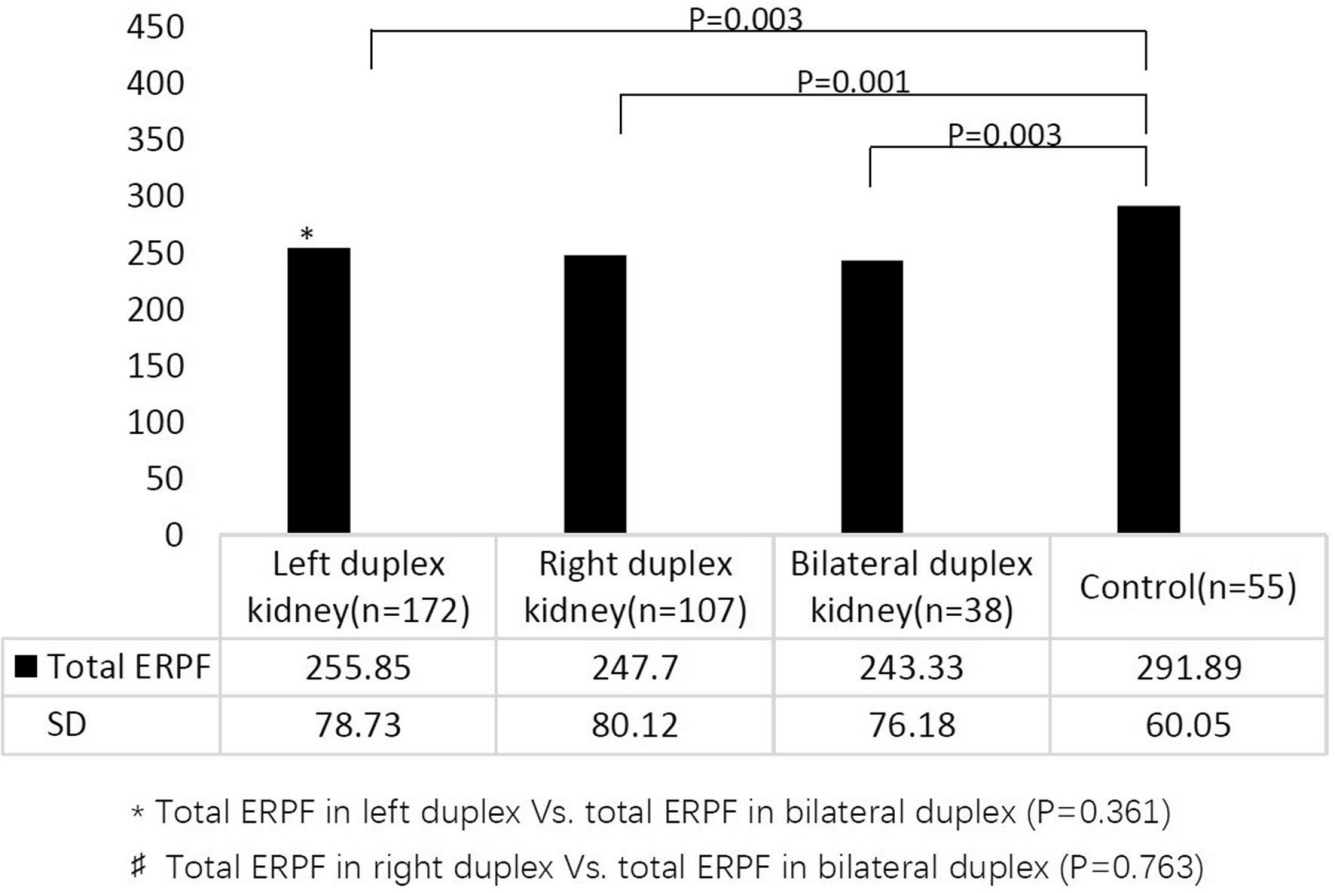
Total renal function in duplex kidney. *ERPF* effective renal plasma flow

The total ERPF values in the left duplex kidney, right duplex kidney, bilateral duplex kidney, and control group were 255.85 ± 78.73 mL/min/m^2^, 247.70 ± 80.12 mL/min/m^2^, 243.33 ± 76.18 mL/min/m^2^, and 291.89 ± 60.05 mL/min/m^2^, respectively. Compared with that in the control group, the total ERPF value in children with left duplex, right duplex, and bilateral duplex kidneys decreased significantly (*P* = 0.003, *P* = 0.001, and *P* = 0.003, respectively) (Fig. 2). The total ERPF value of the bilateral duplex kidney was not different from that of the left and right duplex kidneys (*P* = 0.361 and *P* = 0.763, respectively) (Fig. 2).

### Split renal function in duplex kidneys

**Fig. 3:**
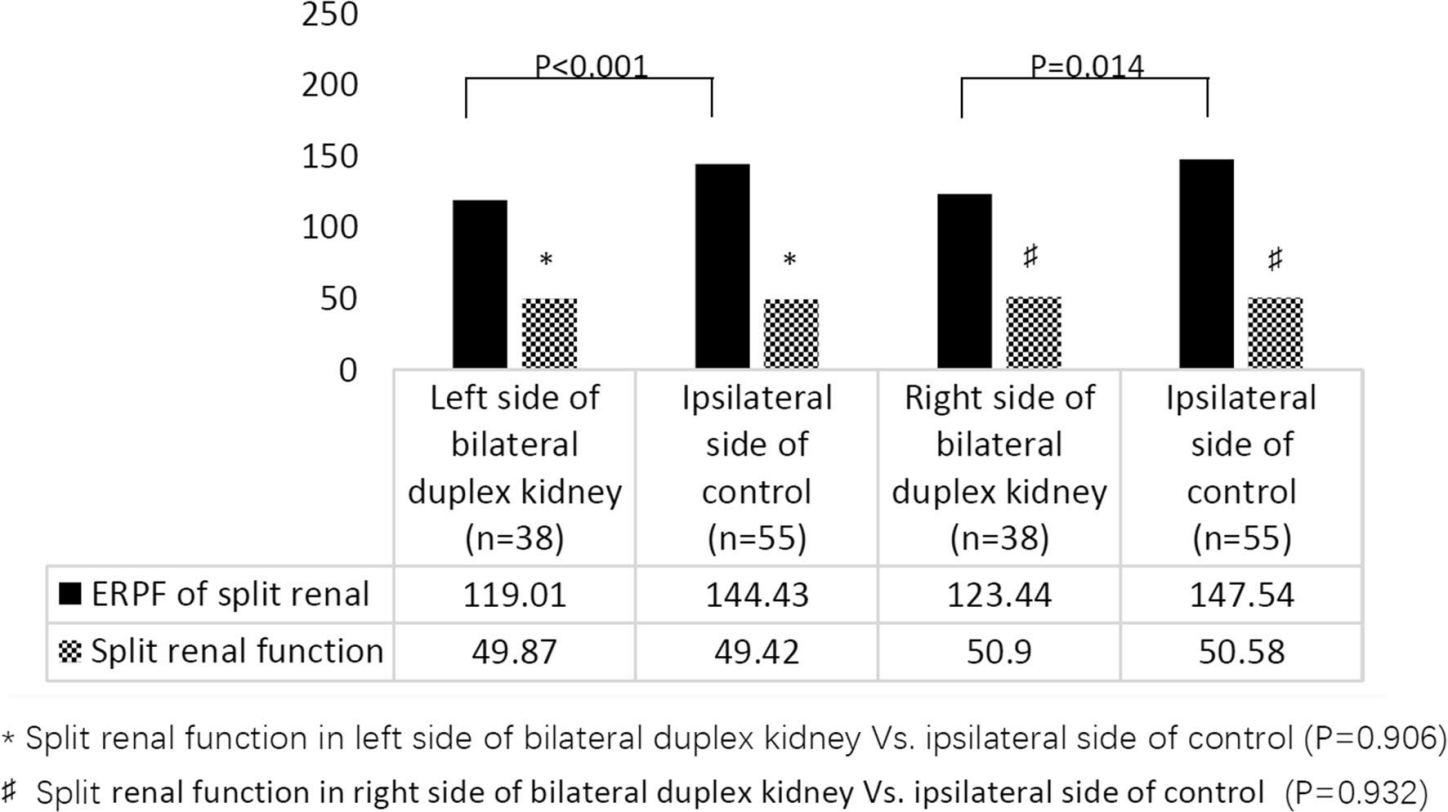
Split renal function in bilateral duplex kidney. *DMSA* dimercaptosuccinic acid, *ERPF* effective renal plasma flow

DMSA scans showed that split renal function in unilateral duplex kidneys (45.58 ± 12.85%) was much lower than that in duplex kidneys on the contralateral side (56.33 ± 11.90%) and ipsilateral side of controls (50.00 ± 11.38%) (*P* < 0.001 and *P* = 0.014, respectively) (Table 2). Split renal function in unilateral duplex kidneys was much higher than that in reflux kidneys (39.52 ± 11.93%) (*P* = 0.001) (Table 2). Split renal function in the contralateral duplex kidney was higher than that in the ipsilateral kidney in the control group (*P* = 0.001). Both left and right split renal functions in bilateral duplex kidneys were similar to those in the control group (*P* = 0.906 and *P* = 0.932, respectively). However, the ERPF values in both the left and right sides of the bilateral duplex kidneys were lower than those in the control ipsilateral duplex kidney (*P* < 0.001 and *P* = 0.014, respectively) (Table 1, Fig. 3).

The ERPF value (split renal function) of the unilateral duplex kidney was 106.70 ± 48.05 mL/min/m^2^ (42.21 ± 13.39%), which was lower than that in the contralateral kidney of controls, 150.18 ± 49.01 mL/min/m^2^ (59.94 ± 10.28%) (P < 0.001). The ERPF value in control ipsilateral duplex kidneys was 145.98 ± 41.16 mL/min/m^2^ (50.01 ± 10.44%), which was higher than that in unilateral duplex kidneys (*P* < 0.001). The ERPF value in the contralateral duplex kidney was slightly higher than that in the ipsilateral kidney in the control group, but the difference was not significant (*P* = 0.127) (Table 2).

## Discussion

A duplex kidney is a common congenital deformity of the kidney and ureter, with an incidence of approximately 0.7%, consisting of approximately 80% unilateral duplication, with slightly more occurrence on the left side than on the right side, and the incidence in girls is approximately twice that in boys [1]. The duplex kidney is usually characterized by dominant development of the lower moiety and hypoplasia of the upper moiety, with poor renal function and a small size, often accompanied by varying degrees of hydro-nephrosis. Renal dysplasia and severe hydro-nephrosis cause renal function damage. The upper moiety of the duplex kidney is often accompanied by ectopic ureteral openings or cysts, whereas the lower duplex kidney is sometimes accompanied by obstruction of the pyeloureteral junction or VUR. The ratio of ureteroceles showed no significant differences among the left or right unilateral and bilateral groups (Table 1). The ectopic ureteral opening was more common in unilateral duplex kidneys than in bilateral duplex ones (Table 1). An ectopic ureteral opening can lead to repeated UTIs, which can cause renal scarring, further damaging kidney function. History of UTI showed no significant differences among the unilateral, bilateral, and control groups (Table 1).

Duplex kidney malformations are usually associated with upper moiety renal dysplasia on the affected side and VUR. VUR is often complicated by recurrent UTIs. Recurrent UTIs and VUR can lead to renal scar formation and damage renal function.

The clinical data of 317 children with duplex kidneys, 55 children with UTIs, and 104 children with primary unilateral VUR were reviewed. The total ratio of VUR in duplex kidneys was 32.35%. The ratios of moderate and severe VUR grades in the unilateral duplex kidney were significantly higher than those in the contralateral kidney (Tables 1, 2). The renal scarring score in unilateral duplex kidney was also higher than that in contralateral duplex kidney. Duplex kidneys are often accompanied by a higher grade of VUR and renal scarring. VUR was more common in reflux kidneys (100%) than in duplex kidneys. The split renal scarring score in the reflux duplex kidney (1.18 ± 1.00) was higher than that in the duplex kidney (0.91 ± 0.93) (*P* = 0.035). The more common VUR was often associated with a high renal scarring score, and renal scarring impaired split renal function.

The renal function of duplex kidneys decreases to varying degrees under the dual damage of primary renal dysplasia and secondary UTI, and renal function must be accurately evaluated. Serum creatinine level is a common indicator of renal function in children, and the glomerular filtration rate (GFR) can be estimated using the Schwartz formula. Serum creatinine levels in the left or right unilateral and bilateral duplex kidneys were similar to those in the control group (*P* = 0.405). Elevation of serum creatinine level was not obvious in this study. Serum creatinine levels cannot reflect changes in renal function in children with duplex kidneys. Serum creatinine level is affected by muscle mass, diet, race, sex and age. Creatinine level change is delayed in response to kidney impairment. Serum cystatin C levels were higher in the bilateral duplex kidney malformation group than in the control group. Cystatin C is a protease inhibitor produced in all nucleated cells. It is produced at a relatively constant rate and released into the plasma. Cystatin C is freely filtered and then catabolized by renal tubular cells. Therefore, the plasma concentration of cystatin C depends primarily on GFR. Cystatin C is considered to be a good biomarker of decreased kidney function. The diagnostic accuracy Cystatin C is comparable or superior to that of serum creatinine level in the discrimination of normal from impaired duplex kidney function [9, 10].

DMSA renal scintigraphy provides an accurate assessment of split renal function [4]. A range of 51–57% can be used as the limit for the normality of the relative function of a unilateral duplex kidney [11]. The DMSA scans showed that split renal function in unilateral duplex kidneys (45.58 ± 12.85%) was much lower than that in duplex kidneys on the contralateral side (56.33 ± 11.90%) and in controls (50.00 ± 11.38%) (*P* < 0.001 and *P* = 0.014, respectively). Split renal function in unilateral duplex kidneys was much higher than that in reflux kidneys (39.52 ± 11.93%). Both left and right split renal functions in bilateral duplex kidneys were similar to those in the control group (*P* = 0.906 and *P* = 0.932, respectively). However, the total ERPF in the bilateral duplex kidney was significantly lower than that in the control group (*P* = 0.003). Notably, evaluation of split renal function in bilateral duplex kidneys should be performed cautiously.

Renal dynamic imaging combines renal morphology imaging with blood perfusion and functional examination to determine renal function with good sensitivity and accuracy [12]. Renal function was assessed using radionuclide nephrography. Renal dynamic imaging is performed using ^99m^Tc-EC, which is a tubule-secreted renal imaging agent that reflects renal arterial blood perfusion and urinary excretion, and the reaction of ^99m^Tc-diethylene-triamine-pentaacetic acid (^99m^Tc-DTPA) is closely related to glomerular filtration function [13]. ERPF measured by ^99m^Tc-EC in renal dynamic imaging can also be used to monitor the long-term prognosis of transplanted kidneys [14]. The normal range of a split renal function is 42–58% [15]. The ERPF values of unilateral duplex kidneys (106.70 ± 48.05 mL/min/m^2^) were significantly lower than those of the contralateral-side kidneys (150.18 ± 49.01 mL/min/m^2^), indicating that unilateral duplex kidneys significantly affected renal function. The ERPF value in the contralateral unilateral duplex kidney was higher than that in the control ipsilateral kidney. This supported that the contralateral kidney of the unilateral duplex kidney had a partial compensative ability. The total ERPF in the left and right unilateral duplex kidneys was similar. The total ERPF in the left and right unilateral duplex kidneys was slightly lower than that in the control group (*P* = 0.003 and *P* = 0.001, respectively). Compared with that of the control group, the total ERPF value of the bilateral duplex kidney decreased significantly. ^99m^Tc-EC dynamic renal imaging and ERPF determination can help evaluate split renal function on both sides of the duplex kidney.

This study screened 317 duplex kidneys at a tertiary center, and the outcomes were objectively measured by professional physicians. Nevertheless, owing to the retrospective design of the study, we could not draw a causal conclusion. Additionally, selection bias for a single-center study with measurement bias cannot be excluded. Residual confounders, such as socioeconomic factors, may have introduced study bias. Owing to the short-term follow-up and single-center study design, the generalization of our conclusions might be limited. Our findings warrant further study, with the need for a well-designed, large-scale, long-term follow-up prospective study.

Although duplex kidney ureteral deformity occurs, renal dysplasia, bladder ureter reflux, VUR, and recurrent UTIs cause kidney scar formation. Risk factors include the a certain degree of decline in the side kidney ERPF value, a certain compensatory ability of the contralateral kidney of the unilateral kidney with ureteral repetitive malformations, and a slight decline in the total ERPF value compared with that of the control group. The results indicated that unilateral duplex kidneys have a limited influence on renal function and good prognosis. The ERPF value of the children with bilateral duplex kidneys was significantly lower than that of the control group, which requires careful follow-up.
